# Supplementary material for: Hippocampal and prefrontal processing of network topology to simulate the future
Source: Nat Commun. 2017 Mar 21;8:14652. doi: 10.1038/ncomms14652 (PMC5364395; doi:10.1038/ncomms14652)
Supplement: Supplementary Information — Supplementary Figures and Supplementary Tables [file ncomms14652-s1.pdf]

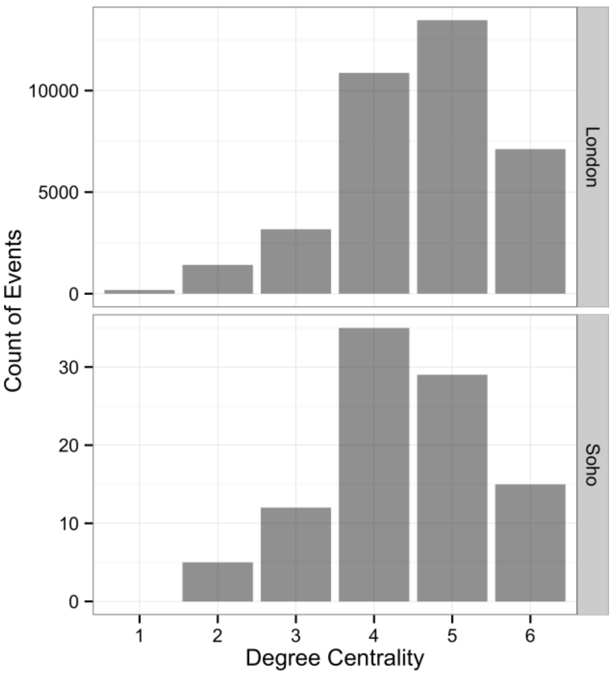

A

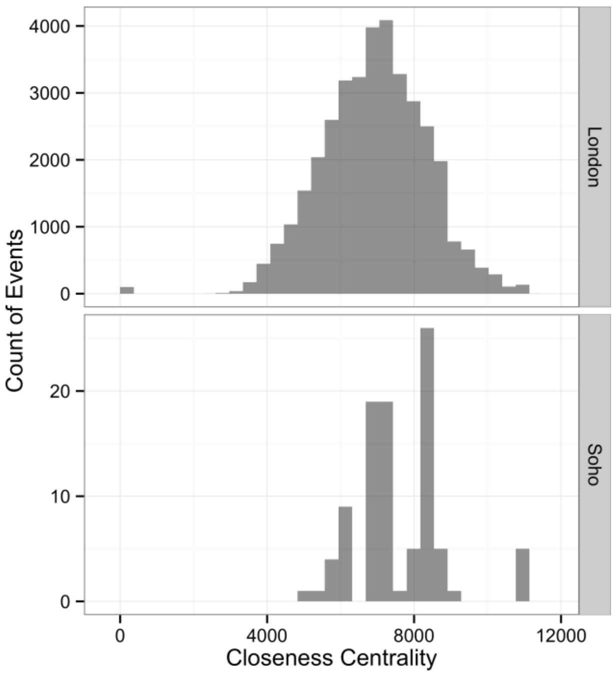

B

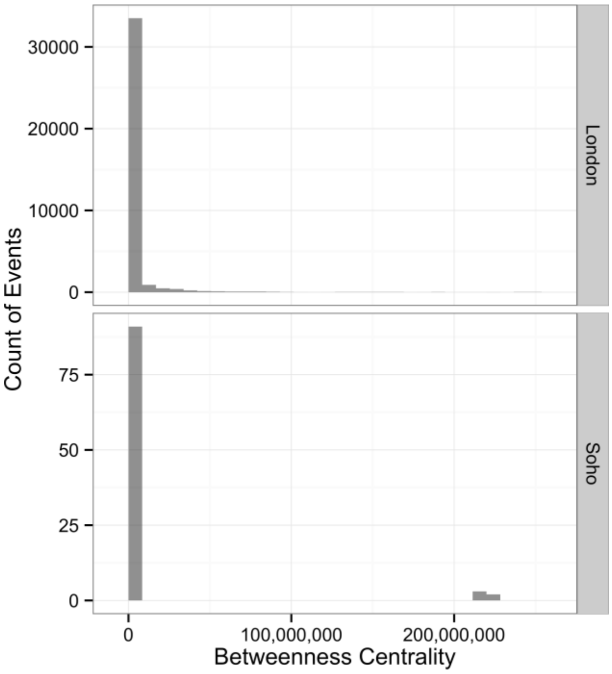

C

Figure 1. Comparison of the frequency of centrality values for central London and our region of Soho studied. The comparison shows that Soho falls well within the distribution for London's streets. While degree centrality (A) and Betweenness centrality (C) did not differ significantly between London and Soho (nonparametric Independent Samples Mann-Whitney U Test non-significant FDR difference $p = 0.041$, Cohen's d effect size 0.18 for Degree, and $p = 0.25$, Cohen's d effect size 0.52 for Betweenness), closeness centrality (B) was skewed to the higher range in Soho (Mann-Whitney U Test significant FDR difference $p < 0.001$, Cohen's d effect size 0.49).

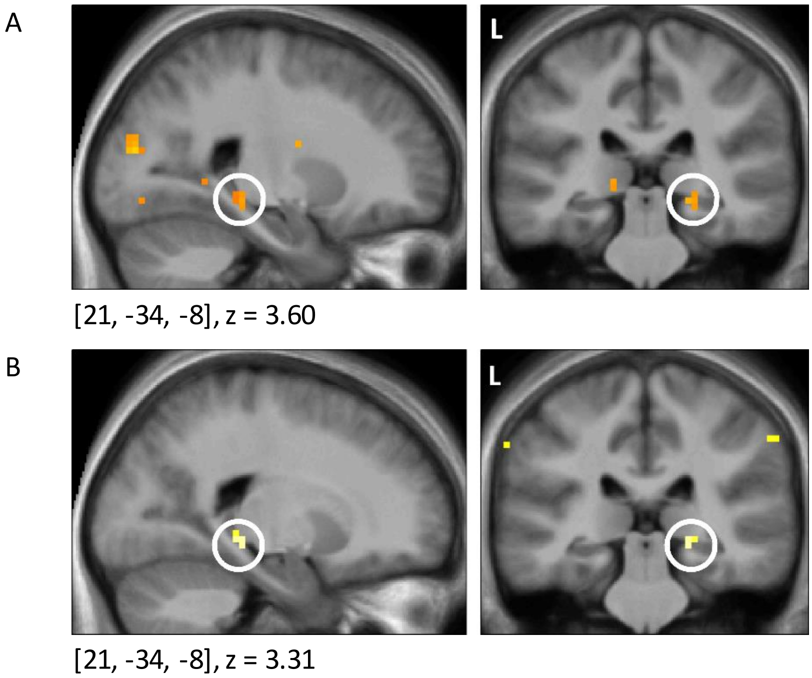

Figure 2. Right posterior hippocampal activity is correlated with the change in degree centrality. (A) Right posterior hippocampal activity significantly correlated ($p < 0.05$ family-wise error (FWE) corrected for a priori regions of interest (ROI)) with the change in degree centrality at Street Entry events during navigation routes in a model containing three parametric modulators of the categorical change in degree centrality, closeness centrality and betweenness centrality. (B) Right posterior hippocampal activity significantly more correlated ($p < 0.05$ FWE for ROI) with the change in degree centrality at Street Entry events for Navigation > Control in the model used for (A). Statistical parametric maps are displayed on the mean structural image at a threshold of $p < 0.005$ uncorrected and 5 voxels minimum cluster size, see **Supplementary Table 3** for details.

A

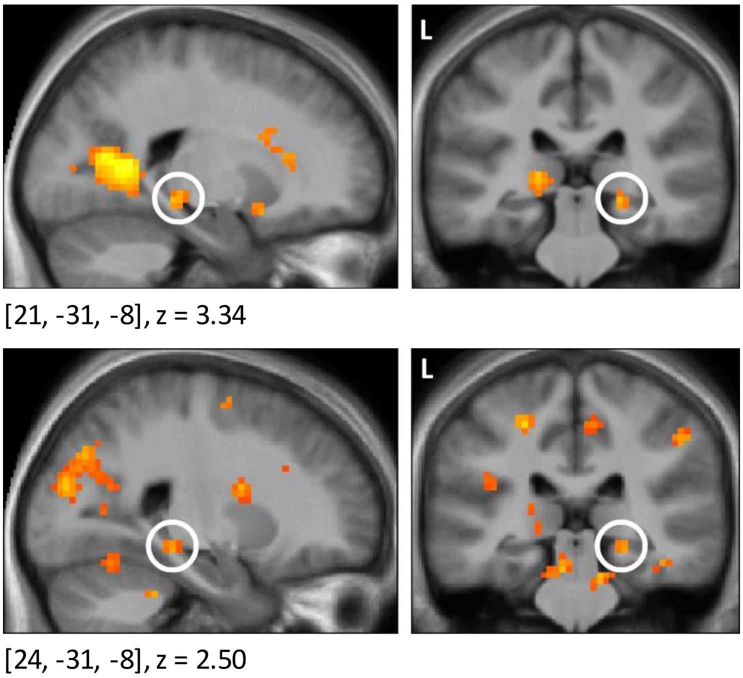

Figure 3. Comparison of correlation of hippocampal activity with degree, closeness and betweenness centrality. (A) Posterior-hippocampus activity for degree centrality > closeness centrality. The activation map is displayed on the mean structural image at a threshold of $p < 0.005$ uncorrected and 5 voxels minimum cluster size. (B) Posterior-hippocampus activity for degree centrality > betweenness centrality. The activation map is displayed on the mean structural image at a threshold of $p < 0.05$ uncorrected and 5 voxels minimum cluster size. See **Supplementary Table 3** for details.

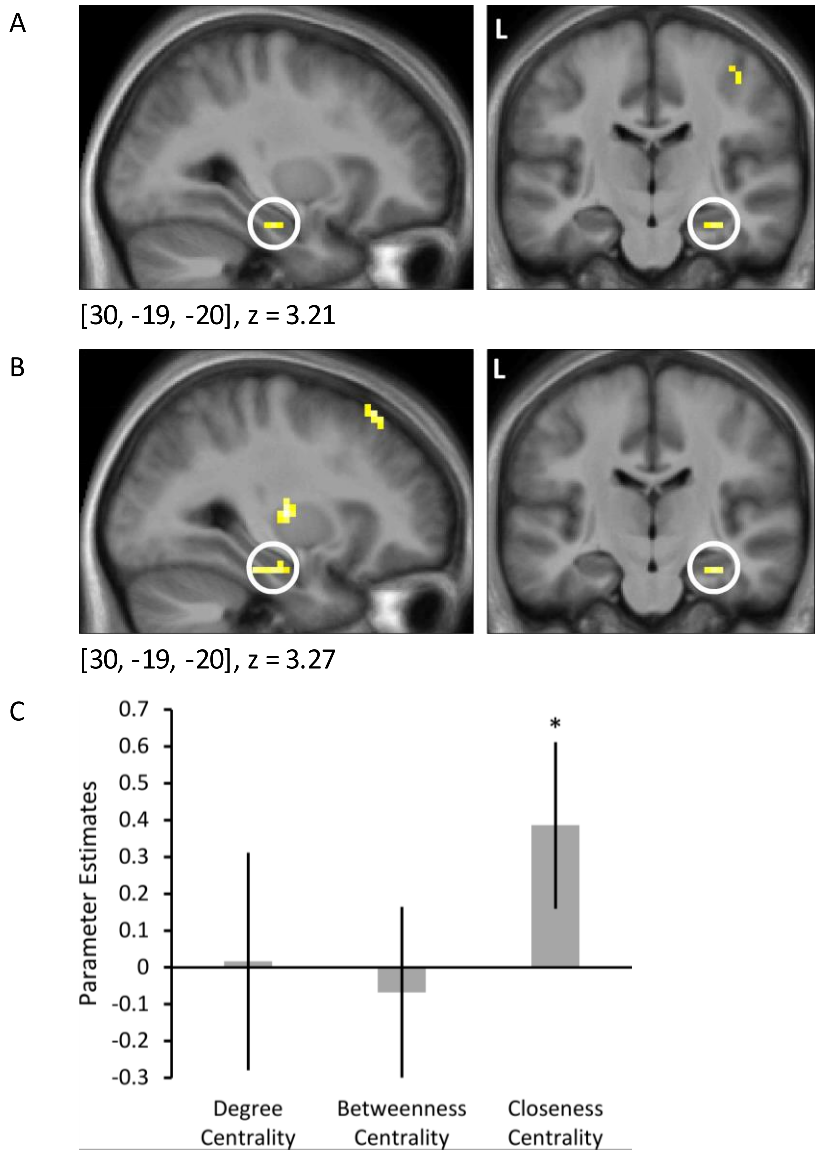

Figure 4. Right anterior hippocampal activity is correlated with the change in closeness centrality. (A) For Navigation routes ($p < 0.05$ family-wise error (FWE) corrected for a priori regions of interest (ROI)), (B) Navigation > Control routes at Street Entry events in a model containing three parametric modulators of the categorical change in degree centrality, closeness centrality and betweenness centrality ($p < 0.05$ FWE for ROI). The activation maps are displayed on the mean structural image at a threshold of $p < 0.005$ uncorrected and 5 voxels minimum cluster size, see **Supplementary Table 3** for details. Follow up control analysis: Because the change in closeness centrality was correlated with the length of the line of sight in the street segment, and with the step depth to boundary (**Supplementary Table 6**) we included these two parameters in new models to determine if the response shown in A and B was significant when controlling for them. We found no significant hippocampal activity in either model (even at a low threshold of $p < 0.01$ uncorrected), nor was anterior hippocampus significantly correlated with the line of sight, or with step depth to boundary ($p < 0.05$ corrected). (C) Parameter estimates for mean activity in the right anterior hippocampus ROI for Navigation > Control condition for a model containing degree centrality ($t_{23} = 0.05$, $p = 0.95$), betweenness centrality ($t_{23} = 0.28$, $p = 0.77$) and closeness centrality ($t_{23} = 2.51$, $p = 0.01$) measures (* = significant at $p < 0.05$, see **Supplementary Table 3** for details). Error bars denote the SEM. Note:

contrasts between these measures (e.g. change in degree centrality > change in closeness centrality) were conducted in the SPM framework - no significant activations were observed in the anterior hippocampus for these contrasts, see **Supplementary Table 3**.

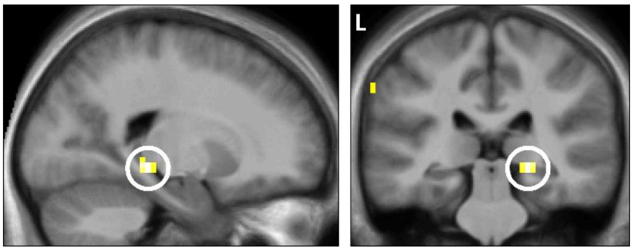

Visible Junctions, z = 3.75

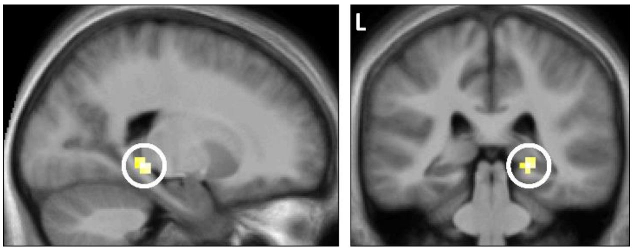

Visible Connecting Streets, z = 3.60

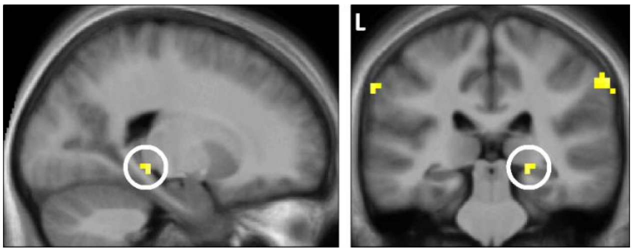

Δ Path Distance to Goal, z = 2.66

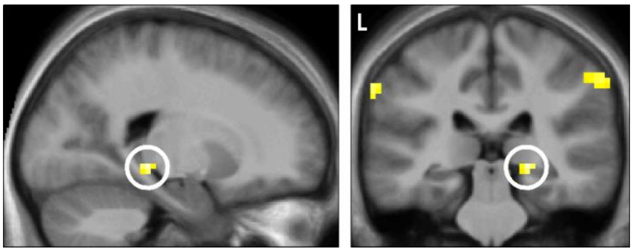

Euclidean Distance to Goal, z = 3.21

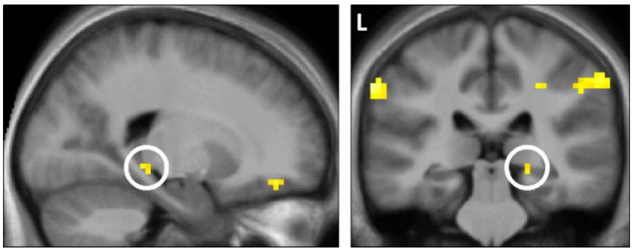

Step Depth to Goal[†], z = 2.50

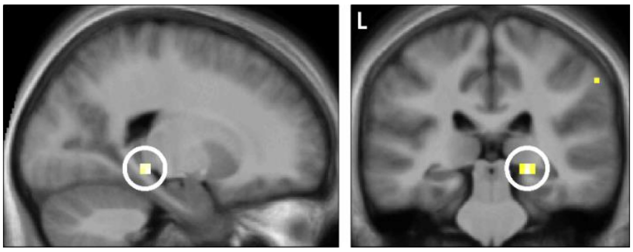

Step Depth to Boundary, z = 3.10

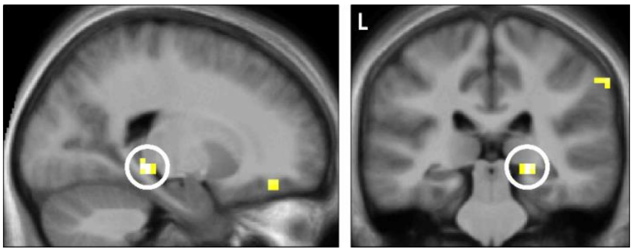

Line of Sight, z = 3.24

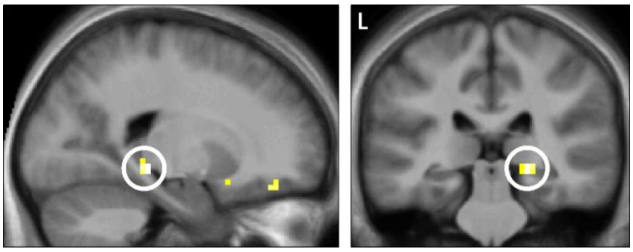

Street Width, z = 3.29

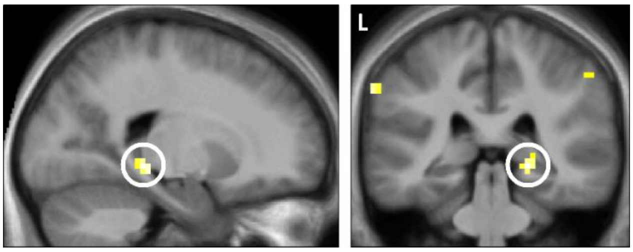

Street Length, z = 3.26

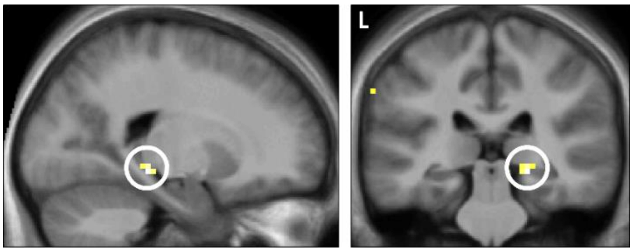

Presence of Visible People, z = 3.02

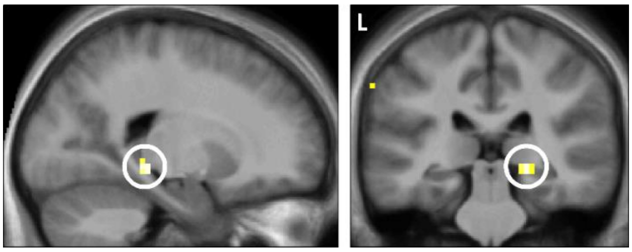

Presence of Visible Vehicles, z = 3.22

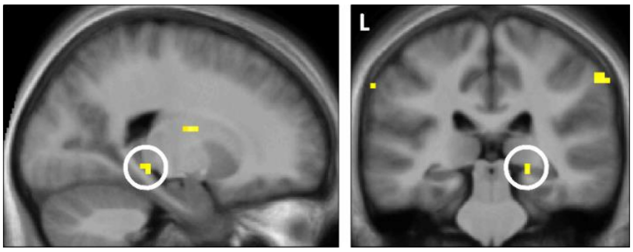

Presence of Visible Shops[†], z = 2.54

Figure 5. Hippocampal activity correlated with the change in degree centrality at Street Entry events accounting for other environmental variables. Correlation of activity of the right posterior hippocampus with categorical change in degree centrality in a contrast comparing Navigation > control conditions for models comprising combination of categorical change in degree centrality and categorical change in other metrics extracted from our analysis of the environment. Statistical parametric maps are displayed on the mean structural image at a threshold of $p < 0.005$ uncorrected and 5 voxels minimum cluster size or at a display threshold of $p < 0.01$ uncorrected[†].

A

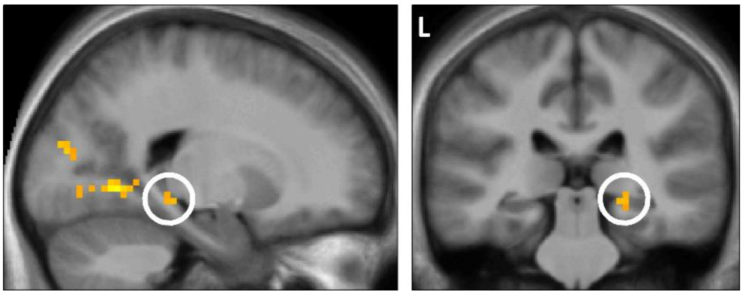

[21, -31, -8], z = 2.83

B

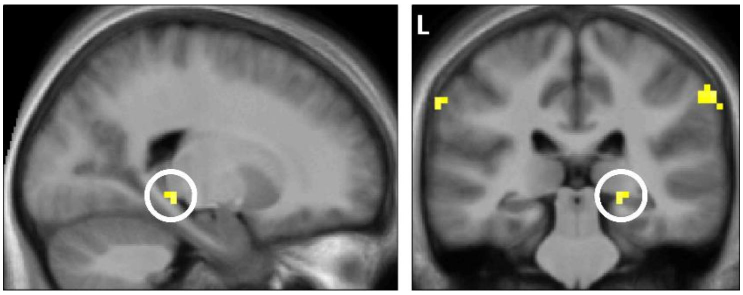

[21, -31, -8], z = 2.66

Figure 6. Hippocampal response to change in degree centrality is independent of responses to the change in path distance at Detours. Correlation of activity of the right posterior hippocampus with categorical change in degree centrality in a model containing the categorical change in degree centrality and the change in path distance in (A) the contrast for Navigation condition and (B) the contrast comparing Navigation > Control tasks. Statistical parametric maps are displayed on the mean structural image at a threshold of $p < 0.005$ (A) and $p < 0.01$ (B) uncorrected and 5 voxels minimum cluster size. See **Supplementary Table 9** for details of the results.

A

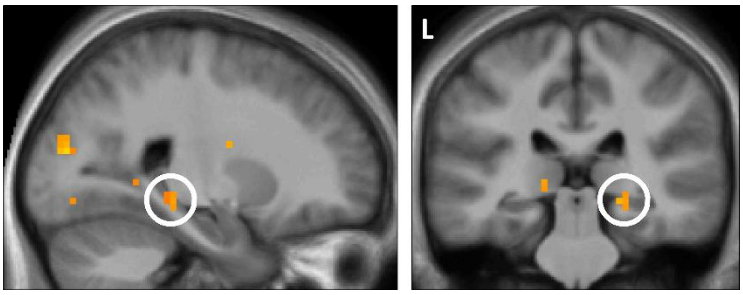

[21, -31, -8], z = 2.80

B

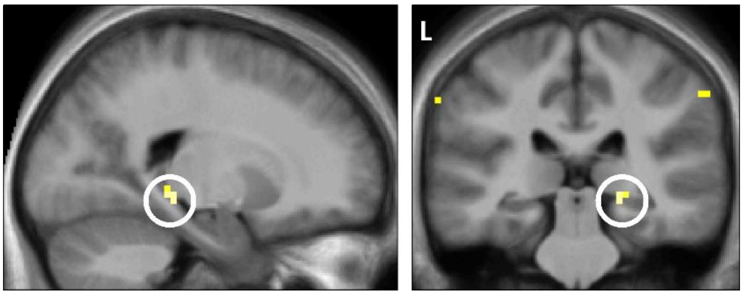

[21, -31, -5], z = 2.88

Figure 7. Hippocampal activity is correlated with the change in degree centrality for the subset of Street Entry events where the degree centrality either increases or decreases. (A) For Navigation routes (B) Navigation > Control routes. The activation maps are displayed on the mean structural image at a threshold of $p < 0.01$ uncorrected and 5 voxels minimum cluster size.

Table 1. Correlation of degree, betweenness and closeness centrality values in Soho

	Degree Centrality		Betweenness Centrality		Closeness Centrality	
	r	*p*	*r*	*p*	*r*	*p*
Raw						
Degree Centrality			-0.163	0.229	0.431	0.001*
Betweenness Centrality	-0.163	0.229			0.028	0.84
Closeness Centrality	0.431	0.001*	0.028	0.840		
Change						
Degree Centrality			-0.095	0.485	0.261	0.052
Betweenness Centrality	-0.095	0.485			0.201	0.138
Closeness Centrality	0.261	0.052	0.201	0.138		

* $p < 0.01$

Table 2. Results from three models examining parametric modulation of either degree, betweenness or closeness centrality *categorical change* values at Street Entry events within Navigation routes (Nav) and for Navigation > Control routes (Con)

					MNI			
Contrast	Brain Area	K_E	T	Z	x	y	z	
Degree Centrality Nav	R Post Hippocampus	16	4.64	3.86	21	-34	-5	*
	R Cuneus	593	7.58	5.31	12	-85	10	
	L Parahippocampus	79	5.17	4.17	-21	-61	-2	
	L Cerebellum	118	5.11	4.14	-36	-70	-17	
	L Precentral Gyrus	7	4.71	3.90	-36	-4	40	
	L Sup Frontal Gyrus	18	4.57	3.82	-24	17	49	
	R Cerebellum	32	4.24	3.61	39	-64	-17	
	L Mid Occipital Gyrus	7	3.97	3.43	48	-70	7	
	L Mid Temporal Gyrus	5	3.93	3.40	42	-79	19	
Degree Centrality Nav > Con	R Post Hippocampus	10	4.25	3.61	21	-34	-5	*
	Thalamus	10	4.49	3.76	3	-19	10	
	L Mid Frontal Gyrus	6	4.18	3.57	-27	17	49	
Betweenness Centrality Nav	R Lingual Gyrus	407	5.68	4.44	15	-82	7	
	R Claustrum	5	4.71	3.90	42	-16	-11	
	L Lingual Gyrus	9	4.70	3.90	-33	-58	4	
	R Sup Frontal Gyrus	14	4.53	3.79	18	50	22	
	R Inf Frontal Gyrus	25	4.43	3.73	48	44	-5	
	R Sup Temporal Gyr	37	4.43	3.73	57	-25	-2	
	R Mid Frontal Gyrus	10	4.35	3.68	42	8	58	
	L Inf Occipital Gyrus	36	4.20	3.58	-42	-88	-5	
	R Inf Frontal Gyrus	5	3.94	3.41	36	35	10	
Betweenness Centrality Nav > Con	R Mid Frontal Gyrus	23	4.65	3.87	39	2	61	
	Thalamus	5	4.31	3.65	0	-19	13	
	L Mid Occipital Gyr	6	4.29	3.64	-45	-82	-8	
	R Inf Frontal Gyrus	5	3.67	3.22	45	47	-5	
Closeness Centrality Nav	R Inf Occipital Gyr	177	6.71	4.94	48	-76	1	
	L Lingual Gyrus	43	6.08	4.64	-33	-61	7	
	R Lingual Gyrus	208	5.38	4.29	9	-91	10	
	L Lingual Gyrus	42	5.18	4.18	-9	-85	-8	
	L Sup Frontal Gyrus	21	4.90	4.01	-9	35	58	
	R Mid Frontal Gyrus	40	4.72	3.91	51	41	-11	
	R Sup Temporal Gyr	12	4.71	3.90	54	5	-5	
	R Paracentral Lobule	7	4.47	3.75	9	-7	46	
	R Sup Frontal Gyrus	18	4.38	3.70	12	35	58	
	R Mid Frontal Gyrus	17	4.28	3.64	39	53	-8	
	R Sup Temporal Gyr	27	4.24	3.61	66	-28	1	
	Med Frontal Gyrus	15	4.20	3.58	3	56	31	
	R Sup Frontal Gyrus	14	3.93	3.40	15	56	22	
	R Mid Frontal Gyrus	5	3.82	3.33	39	29	46	
Closeness Centrality Nav > Con	R Mid Frontal Gyrus	72	5.30	4.24	45	8	52	
	L Inf Frontal Gyrus	59	5.27	4.22	-54	35	1	
	R Inf Frontal Gyrus	94	5.11	4.14	45	47	-5	
	R Fusiform Gyrus	15	4.44	3.73	54	-61	-14	
	Posterior Cingulate	6	4.39	3.71	-3	-46	19	
	R Sup Temporal Gyr	17	4.24	3.61	54	-52	22	
	L Sup Frontal Gyrus	8	4.23	3.60	-9	35	58	

	R Mid Frontal Gyrus	5	3.94	3.44	60	23	22
	L Sup Temporal Gyr	5	3.82	3.33	-57	-28	1
	R Sup Frontal Gyrus	9	3.72	3.26	12	35	58

* = Significant at $p < 0.05$ corrected for region of interest, 5 voxel minimum cluster size (refer to **Methods** for further details), otherwise significant at a threshold of $p < 0.001$ uncorrected, 5 voxel minimum cluster size, provided for completeness. No regions survived at a threshold of $p < 0.05$, 5 voxel minimum cluster size, family-wise error corrected for whole brain volume. R = Right, L = Left, Post = Posterior, Med = Medial, Inf = Inferior, Sup = Superior, Mid = Middle, Gyr = Gyrus.

Table 3. Same analysis as Table 2 above (examining categorical change), except a single statistical model contains all three spatial parameters

Contrast	Brain Area	K_E	T	Z	MNI x	y	z	
Degree Centrality Nav	R Post Hippocampus	6	4.23	3.60	21	-34	-8	*
	L Lingual Gyrus	64	6.77	4.97	-18	-61	-2	
	R Cuneus	207	6.60	4.90	9	-82	10	
	L Mid Occipital Gyrus	102	4.53	3.79	-36	-70	1	
	L Insula	8	4.18	3.57	-33	-10	19	
	L Sup Frontal Gyrus	10	4.07	3.50	-33	53	28	
	R Fusiform Gyrus	9	4.03	3.47	39	-76	-8	
	R Mid Occipital Gyr	18	3.98	3.44	54	-73	4	
	R Lingual Gyrus	9	3.90	3.38	15	-52	1	
Degree Centrality Nav > Con	R Post Hippocampus	6	3.79	3.31	21	-34	-8	*
Betweenness Centrality Nav	R Inf Frontal Gyrus	5	4.20	3.58	54	44	-2	
Betweenness Centrality Nav > Con	-	-	-	-	-	-	-	
Closeness Centrality Nav †	R Ant Hippocampus	6	3.65	3.21	30	-19	-20	*
	R Mid Occipital Gyr	33	4.54	3.80	51	-70	7	
	L Mid Occipital Gyr	7	3.82	3.33	-36	-70	10	
Closeness Centrality Nav > Con †	R Ant Hippocampus	11	3.73	3.27	30	-19	-20	*
	L Inf Frontal Gyrus	9	4.21	3.59	-54	32	10	
	R Mid Frontal Gyrus	8	3.96	3.42	45	17	52	
Nav Degree > Betweenness Cen	L Lingual Gyrus	21	5.01	4.08	-21	-67	-2	
	R Mid Occipital Gyr	20	4.58	3.82	54	-73	7	
	L Precuneus	10	4.58	3.82	-18	-70	22	
	L Cuneus	15	3.98	3.44	-12	-76	34	
	L Precuneus	5	3.64	3.20	-24	-40	49	
Nav Degree > Closeness Cen	R Post Hippocampus	23	3.84	3.34	21	-31	-8	*
	L Thalamus	515	7.01	5.08	-18	-34	4	
	L Fusiform Gyrus	193	5.82	4.52	-36	-55	-8	
	R Dentate	9	4.46	3.75	15	-64	-26	
	L Caudate	8	4.24	3.61	-6	17	13	
	L Claustrum	7	4.14	3.54	-27	29	-2	
	R Caudate	10	4.02	3.46	15	23	13	
Nav Betweenness > Degree Cen	-	-	-	-	-	-	-	
Nav Betweenness > Closeness Cen	L Caudate	7	4.34	3.67	-36	-16	-11	
Nav Closeness > Degree Cen	-	-	-	-	-	-	-	
Nav Closeness > Betweenness Cen	Mid Occipital Gyrus	8	4.12	3.53	51	-70	7	

* = Significant at $p < 0.05$ corrected for region of interest, 5 voxel minimum cluster size (refer to **Methods** for further details, see also **Supplementary Fig. 2**), otherwise significant at a threshold of $p < 0.001$ uncorrected, 5 voxel minimum cluster size, provided for completeness. No regions survived at a threshold of $p < 0.05$ family-wise error corrected for whole brain volume, 5 voxel minimum cluster size. R = Right, L = Left, Post = Posterior, Med = Medial, Inf = Inferior, Sup = Superior, Mid = Middle, Gyr = Gyrus, Cen = Centrality. † Right anterior hippocampus was not significantly correlated with closeness centrality in the model in which closeness centrality was included alone, see **Supplementary Table 2** and **Supplementary Fig. 4**.

Table 4. Results from a similar analysis to that used for Table 2 except *raw* centrality measures were examined rather than the categorical change.

Contrast	Brain Area	K_E	T	Z	MNI x	y	z
Degree Centrality Nav	-						
Degree Centrality Nav > Con	-						
Betweenness Centrality Nav	Med Frontal Gyrus	126	5.35	4.27	9	59	31
	R Inf Frontal Gyrus	78	5.21	4.19	48	32	-2
	L Sup Temporal Gyr	17	4.82	3.97	-63	-52	22
	R Inf Frontal Gyrus	12	4.33	3.67	60	23	16
	R Mid Frontal Gyrus	5	4.24	3.61	33	8	49
	R Supramarginal Gyr	48	4.18	3.57	63	-49	34
	L Angular Gyrus	27	4.17	3.56	-51	-61	37
	R Mid Occipital Gyr	9	4.06	3.49	30	-94	16
	R Mid Temporal Gyr	6	3.85	3.35	66	-43	1
Betweenness Centrality Nav > Con	-	-	-	-	-	-	-
Closeness Centrality Nav	R Precentral Gyrus	42	4.87	3.99	51	20	4
	R Supramarginal Gyr	48	4.73	3.91	57	-52	22
	L Sup Temporal Gyr	16	4.33	3.67	-66	-49	22
Closeness Centrality Nav > Con	L Inf Frontal Gyrus	12	3.71	3.25	-54	35	4

Significant at a threshold of $p < 0.001$ uncorrected, 5 voxel minimum cluster size, provided for completeness. No regions survived at a threshold of $p < 0.05$, 5 voxel minimum cluster size, family-wise error corrected for whole brain volume, or correcting for our hippocampal ROI. R = Right, L = Left, Post = Posterior, Med = Medial, Inf = Inferior, Sup = Superior, Mid = Middle, Gyr = Gyrus

Table 5. Correlation between raw values of degree, betweenness and closeness centrality and other environmental properties at Street Entry events

	Degree Centrality		Betweenness Centrality		Closeness Centrality	
	r	*p*	*r*	*p*	*r*	*p*
Number of Visible Junctions	0.02	0.83	-0.21	0.10	-0.11	0.40
Number of Visible Connecting St	0.12	0.38	-0.22	0.09	-0.13	0.33
Path Distance to Goal	0.07	0.58	-0.07	0.58	-0.05	0.67
Euclidean Distance to Goal	0.07	0.59	-0.13	0.30	-0.08	0.54
Step Depth to Goal	0.01	0.89	-0.16	0.21	-0.10	0.46
Step Depth to Boundary	-0.18	0.17	-0.17	0.21	-0.29	0.02
Line of Sight	0.46	< 0.001*	0.51	< 0.001*	0.57	< 0.001*
Street Width	0.19	0.15	0.37	0.005*	0.35	0.007*
Street Length	0.19	0.16	-0.05	0.69	0.00	0.97
Presence of Visible People	0.35	0.008*	0.43	0.001*	0.44	0.001*
Presence of Visible Vehicles	0.07	0.57	0.07	0.60	0.17	0.19
Presence of Visible Shops	0.53	< 0.001*	0.18	0.16	0.27	0.03

* false discovery rate (FDR) corrected $p < 0.05$

Table 6. Correlation between the change in degree, betweenness and closeness centrality and the change in other properties of the environment at Street Entry events.

	Degree Centrality		Betweenness Centrality		Closeness Centrality	
	r	*p*	*r*	*p*	*r*	*p*
Number of Visible Junctions	-0.04	0.74	0.14	0.29	0.07	0.60
Number of Visible Connecting St	-0.00	0.98	0.15	0.25	0.02	0.83
Path Distance to Goal	0.07	0.56	0.33	0.01	0.22	0.09
Euclidean Distance to Goal	0.19	0.15	0.18	0.17	0.01	0.89
Step Depth to Goal	0.00	0.98	-0.05	0.66	-0.15	0.26
Step Depth to Boundary	-0.17	0.20	-0.46	< 0.001*	-0.37	0.004*
Line of Sight	0.15	0.25	0.52	< 0.001*	0.60	< 0.001*
Street Width	0.18	0.18	0.47	< 0.001*	0.29	0.02
Street Length	0.06	0.61	0.00	0.95	-0.09	0.48
Presence of Visible People	0.28	0.03	0.16	0.23	0.24	0.06
Presence of Visible Vehicles	0.15	0.26	0.23	0.08	0.36	0.006*
Presence of Visible Shops	0.19	0.14	0.05	0.68	-0.00	0.97

* false discovery rate (FDR) corrected $p < 0.05$

Table 7. Correlation between different properties of the environment at Street Entry events

	Visible Junctions		Visible Connecting St		Path Distance to Goal		Euclidean Distance to Goal	
	r	*p*	*r*	*p*	*r*	*p*	*r*	*p*
Number of Visible Junctions			0.78	0.00*	0.06	0.63	0.28	0.03
Number of Visible Connecting St	0.78	0.00*			0.14	0.28	0.20	0.12
Path Distance to Goal	0.06	0.63	0.14	0.28			0.21	0.11
Euclidean Distance to Goal	0.28	0.03	0.20	0.12	0.21	0.11		
Step Depth to Goal	0.25	0.05	0.09	0.50	0.33	0.01	0.78	0.00*
Step Depth to Boundary	-0.00	0.98	-0.03	0.77	-0.20	0.13	0.05	0.71
Line of Sight	0.12	0.36	0.11	0.39	0.12	0.37	-0.04	0.74
Street Width	-0.02	0.87	-0.0	0.82	0.22	0.08	0.00	0.95
Street Length	-0.19	0.15	-0.10	0.42	0.17	0.21	-0.20	0.13
Presence of Visible People	0.01	0.89	-0.01	0.93	0.07	0.60	-0.02	0.83
Presence of Visible Vehicles	0.19	0.14	0.12	0.34	0.10	0.45	0.02	0.83
Presence of Visible Shops	0.12	0.34	0.33	0.01	-0.03	0.78	-0.13	0.31
	**Step Depth to Goal**		**Step Depth to Boundary**		**Line of Sight**		**Street Width**	
	r	*p*	*r*	*p*	*r*	*p*	*r*	*p*
Number of Visible Junctions	0.25	0.05	-0.00	0.98	0.12	0.36	-0.02	0.87
Number of Visible Connecting St	0.09	0.50	-0.03	0.77	0.11	0.39	-0.03	0.82
Path Distance to Goal	0.33	0.01	-0.20	0.13	0.12	0.37	0.22	0.08
Euclidean Distance to Goal	0.78	0.00*	0.05	0.71	-0.04	0.74	0.00	0.95
Step Depth to Goal			0.13	0.32	-0.10	0.44	0.03	0.81
Step Depth to Boundary	0.13	0.32			-0.32	0.01	-0.30	0.02
Line of Sight	-0.10	0.44	-0.32	0.01			0.59	0.00*
Street Width	0.03	0.81	-0.30	0.02	0.59	0.00*		
Street Length	-0.10	0.43	-0.18	0.18	0.23	0.08	0.35	0.00*
Presence of Visible People	-0.02	0.83	-0.04	0.74	0.37	0.00*	0.26	0.04
Presence of Visible Vehicles	0.08	0.54	-0.05	0.67	0.28	0.03	0.37	0.00*
Presence of Visible Shops	-0.24	0.06	-0.28	0.03	0.2	0.07	0.15	0.25

	Street Length		Visible People		Visible Vehicles		Visible Shops	
	r	*p*	*r*	*p*	*r*	*p*	*r*	*p*
Number of Visible Junctions	-0.19	0.15	0.01	0.89	0.19	0.14	0.12	0.34
Number of Visible Connecting St	-0.10	0.42	-0.01	0.93	0.12	0.34	0.33	0.01
Path Distance to Goal	0.17	0.21	0.07	0.60	0.10	0.45	-0.03	0.78
Euclidean Distance to Goal	-0.20	0.13	-0.02	0.83	0.02	0.83	-0.13	0.31
Step Depth to Goal	-0.10	0.43	-0.02	0.83	0.08	0.54	-0.24	0.06
Step Depth to Boundary	-0.18	0.18	-0.04	0.74	-0.05	0.67	-0.28	0.03
Line of Sight	0.23	0.08	0.37	0.00*	0.28	0.03	0.24	0.07
Street Width	0.35	0.00*	0.26	0.04	0.37	0.00*	0.15	0.25
Street Length			-0.16	0.24	0.18	0.16	0.13	0.33
Presence of Visible People	-0.16	0.24			0.11	0.40	0.25	0.05
Presence of Visible Vehicles	0.18	0.16	0.11	0.40			0.27	0.04
Presence of Visible Shops	0.13	0.33	0.25	0.05	0.27	0.04		

* false discovery rate (FDR) corrected $p < 0.05$

Table 8. Correlation between the *change* in different properties of the environment at Street Entry events.

	Visible Junctions		Visible Connecting St		Path Distance to Goal		Euclidean Distance to Goal	
	r	*p*	*r*	*p*	*r*	*p*	*r*	*p*
Number of Visible Junctions			0.86	0.00*	0.17	0.22	0.00	0.99
Number of Visible Connecting St	0.86	0.00*			0.17	0.22	0.16	0.24
Path Distance to Goal	0.16	0.22	0.16	0.22			0.29	0.03
Euclidean Distance to Goal	0.00	0.99	0.16	0.23	0.29	0.03		
Step Depth to Goal	0.03	0.78	0.01	0.94	0.04	0.80	0.53	0.00*
Step Depth to Boundary	-0.07	0.58	-0.04	0.75	-0.12	0.38	0.01	0.97
Line of Sight	0.21	0.10	0.14	0.29	0.26	0.05	0.09	0.50
Street Width	0.24	0.06	0.12	0.35	0.17	0.20	-0.04	0.77
Street Length	-0.14	0.29	-0.06	0.61	0.23	0.09	0.11	0.43
Presence of Visible People	0.21	0.11	0.17	0.20	0.09	0.49	-0.09	0.50
Presence of Visible Vehicles	0.22	0.09	0.21	0.11	0.07	0.59	0.01	0.97
Presence of Visible Shops	0.26	0.04	0.29	0.02	-0.03	0.80	0.20	0.14

	Step Depth to Goal		Step Depth to Boundary		Line of Sight		Street Width	
	r	*p*	*r*	*p*	*r*	*p*	*r*	*p*
Number of Visible Junctions	0.04	0.78	-0.07	0.59	0.22	0.11	0.25	0.07
Number of Visible Connecting St	0.01	0.94	-0.04	0.76	0.14	0.30	0.13	0.35
Path Distance to Goal	0.04	0.80	-0.12	0.38	0.26	0.05	0.17	0.20
Euclidean Distance to Goal	0.53	0.00*	0.01	0.97	0.09	0.50	-0.04	0.77
Step Depth to Goal			-0.01	0.95	0.11	0.41	0.09	0.52
Step Depth to Boundary	-0.01	0.95			-0.44	0.00*	-0.42	0.00*
Line of Sight	0.11	0.41	-0.44	0.00*			0.49	0.00*
Street Width	0.09	0.52	-0.42	0.00*	0.49	0.00*		
Street Length	0.10	0.45	0.07	0.59	0.12	0.39	0.10	0.48
Presence of Visible People	-0.04	0.75	-0.28	0.04	0.22	0.11	0.18	0.19
Presence of Visible Vehicles	-0.04	0.78	-0.29	0.03	0.33	0.01	0.43	0.00*
Presence of Visible Shops	0.21	0.12	-0.16	0.25	0.03	0.85	0.28	0.03

	Street Length		Visible People		Visible Vehicles		Visible Shops	
	r	*p*	*r*	*p*	*r*	*p*	*r*	*p*
Number of Visible Junctions	-0.14	0.30	0.21	0.12	0.23	0.10	0.27	0.05
Number of Visible Connecting St	-0.07	0.62	0.17	0.21	0.22	0.11	0.29	0.03
Path Distance to Goal	0.23	0.09	0.09	0.49	0.07	0.59	-0.03	0.80
Euclidean Distance to Goal	0.11	0.43	-0.09	0.50	0.01	0.97	0.20	0.14
Step Depth to Goal	0.10	0.45	-0.04	0.75	-0.04	0.78	0.21	0.12
Step Depth to Boundary	0.07	0.59	-0.28	0.04	-0.29	0.03	-0.16	0.25
Line of Sight	0.12	0.39	0.22	0.11	0.33	0.01	0.03	0.85
Street Width	0.10	0.48	0.18	0.19	0.43	0.00*	0.28	0.03
Street Length			0.00	1.00	-0.05	0.70	0.12	0.39
Presence of Visible People	0.00	1.00			0.18	0.18	0.09	0.51
Presence of Visible Vehicles	-0.05	0.70	0.18	0.18			0.44	0.00*
Presence of Visible Shops	0.12	0.39	0.09	0.51	0.44	0.00*		

* false discovery rate (FDR) corrected $p < 0.05$

Table 9. Parametric modulation of categorical change in degree centrality values and change in path distance at Street Entry events after Detour events for Nav and Nav > Con contrasts.

					MNI			
Contrast	Brain Area	K_E	T	Z	x	y	z	
Degree Centrality Nav	R Post Hippocampus	8	3.13	2.83	21	-31	-8	*
	R Lingual Gyrus	126	5.67	4.44	12	-79	7	
Degree Centrality Nav > Con	R Post Hippocampus	5	2.91	2.66	21	-31	-8	**

* = Significant at $p < 0.005$ uncorrected, 5 voxel minimum cluster size (refer to **Methods** for further details, see also **Supplementary Fig. 6**), otherwise significant at a threshold of $p < 0.001$ uncorrected, 5 voxel minimum cluster size, provided for completeness. ** All regions significant at $p < 0.01$ uncorrected, 5 voxel minimum cluster size. No regions survived at a threshold of $p < 0.05$ family-wise error corrected for whole brain volume, 5 voxel minimum cluster size. Nav = Navigation, Con = Control, R = Right, L = Left, Post = Posterior

Table 10. Experiment 2 - Performance of the participants in detecting of changes in the degree centrality at each Street Entry event during viewing the film simulations.

	All Street Entries		Non-zero Street Entries[†]	
	mean (SD)	*p*	mean (SD)	*p*
Naïve (n = 11)	45.60% (11.53)	$p = 0.014$*	39.77% (16.49)	$p = 0.243$
Trained (n = 2)	83.05% (2.39)	$p < 0.001$*	72.91% (8.83)	$p < 0.001$*

[†] only Street Entries with changing degree centrality (40.67% of the entries). * $p < 0.05$ showing Binomial test comparing the performance of the participants with chance level of 33.33% (three possible choices at each decision point). We examined non-zero street entries because the middle response button was for 'no change', which might reasonably be the default response of a naïve participant, which would lead to around 40.67% correct answers (see **Methods)**.

Table 11. Correlation of the left and right prefrontal cortex (PFC) with breath-first search after Detour events within navigation routes (Nav) and for Nav > Control routes (Con)

					MNI		
Contrast	Brain Area	K_E	T	Z	x	y	z
Nav	R Inf Frontal Gyrus	15	4.56	3.81	48	50	1
	L Inf Frontal Gyrus	15	4.25	3.61	-36	23	-5
	R Angular Gyrus	30	5.29	4.24	57	-55	31
	L Angular Gyrus	21	3.82	3.32	-48	-55	40
	R Ant Insular	13	4.62	3.84	30	20	1
	L Sup Frontal Gyrus	13	4.49	3.76	-15	17	55
Nav > Con	R Inf Frontal Gyrus	71	5.45	4.32	51	50	1*
	L Inf Frontal Gyrus	50	4.08	3.50	-45	41	-2*
	L Angular Gyrus	49	4.32	3.66	-54	-61	31

* = Significant at $p < 0.05$ corrected for region of interest, 5 voxel minimum cluster size (refer to **Methods** for further details), otherwise significant at a threshold of $p < 0.001$ uncorrected, 5 voxel minimum cluster size, provided for completeness. No regions survived at a threshold of $p < 0.05$ family-wise error corrected for whole brain volume, 5 voxel minimum cluster size. R = Right, L = Left, Post = Posterior, Med = Medial, Inf = Inferior, Sup = Superior.
